# Effect of Mild Hypothermia on the Coagulation-Fibrinolysis System and Physiological Anticoagulants after Cardiopulmonary Resuscitation in a Porcine Model

**DOI:** 10.1371/journal.pone.0067476

**Published:** 2013-06-20

**Authors:** Ping Gong, Ming-Yue Zhang, Hong Zhao, Zi-Ren Tang, Rong Hua, Xue Mei, Juan Cui, Chun-Sheng Li

**Affiliations:** 1 Department of Emergency, First Hospital affiliated to Dalian Medical University, Dalian, Liaoning, People’s Republic of China; 2 Department of Emergency, Beijing Chaoyang Hospital, Capital Medical University, Beijing, People’s Republic of China; Maastricht University Medical Center, The Netherlands

## Abstract

The aim of this study was to evaluate the effect of mild hypothermia on the coagulation-fibrinolysis system and physiological anticoagulants after cardiopulmonary resuscitation (CPR). A total of 20 male Wuzhishan miniature pigs underwent 8 min of untreated ventricular fibrillation and CPR. Of these, 16 were successfully resuscitated and were randomized into the mild hypothermia group (MH, *n* = 8) or the control normothermia group (CN, *n* = 8). Mild hypothermia (33°C) was induced intravascularly, and this temperature was maintained for 12 h before pigs were actively rewarmed. The CN group received normothermic post-cardiac arrest (CA) care for 72 h. Four animals were in the sham operation group (SO). Blood samples were taken at baseline, and 0.5, 6, 12, 24, and 72 h after ROSC. Whole-body mild hypothermia impaired blood coagulation during cooling, but attenuated blood coagulation impairment at 72 h after ROSC. Mild hypothermia also increased serum levels of physiological anticoagulants, such as PRO C and AT-III during cooling and after rewarming, decreased EPCR and TFPI levels during cooling but not after rewarming, and inhibited fibrinolysis and platelet activation during cooling and after rewarming. Finally, mild hypothermia did not affect coagulation-fibrinolysis, physiological anticoagulants, or platelet activation during rewarming. Thus, our findings indicate that mild hypothermia exerted an anticoagulant effect during cooling, which may have inhibitory effects on microthrombus formation. Furthermore, mild hypothermia inhibited fibrinolysis and platelet activation during cooling and attenuated blood coagulation impairment after rewarming. Slow rewarming had no obvious adverse effects on blood coagulation.

## Introduction

Despite new therapeutic approaches in recent decades, the prognosis after CA and cardiopulmonary resuscitation (CPR) has remained poor. In a recent study of 24,132 patients in the United Kingdom who were admitted to critical care units after CA, the in-hospital mortality rate was 71% [1]. Reperfusion failure, ischemia-reperfusion injury, and cerebral injury may be responsible for an overwhelming systemic inflammatory response associated with elevated plasma cytokines, presence of circulating endotoxin, leukocyte dysregulation, and adrenal dysfunction, reflecting a picture similar to that observed in severe sepsis [2], [3]. Current research has shown that CPR and return of spontaneous circulation (ROSC) are associated with marked activation of blood coagulation without adequate concomitant activation of endogenous fibrinolysis [4], [5]. Methods to attenuate this dysfunction could have great clinical importance.

Recently, two independent groups have shown that induction of mild hypothermia in patients after CA is associated with reduced mortality and a better neurological outcome compared with normothermic controls [6], [7]. Based on these findings, 2010 American Heart Association Guidelines for Cardiopulmonary Resuscitation and Emergency Cardiovascular Care recommend that unconscious, adult patients successfully resuscitated from an out-of-hospital ventricular fibrillation (VF) CA should be cooled to 32–34°C for 12–24 h. Though previous researchers have found that blood coagulation disorders are one of the complications of mild hypothermia therapy and hyperfibrinolysis is common in out-of-hospital CA [8], [9], [10], there are limited data available in the literature regarding the effect of mild hypothermia on the coagulation-fibrinolysis system and physiological anticoagulants from the time of CPR to 72 h after ROSC. This was the subject of our study.

## Materials and Methods

### Animal Preparation

This study was conducted with the approval of the Animal Care and Use Committee at Chaoyang Hospital, affiliated with Capital Medical University, China. The protocol was approved by the Committee on the Ethics of Animal Experiments of Capital Medical University (Permit Number: 2010-D-013). All animals were treated in compliance with the National Research Council’s 1996 Guide for the Care and Use of Laboratory Animals.

Wuzhishan miniature pigs were selected for this study. After 20 generations of inbreeding, Wuzhishan miniature pigs have the highest inbreeding coefficient (more than 0.965), more stable heredity, and less variability between individual animals, as well as a closer resemblance to human beings [11]. Thus, they are considered an appropriate experimental model for CA.

A total of 24 Wuzhishan miniature pigs (12–13 months old, 27.5–33 kg) were fasted overnight but had free access to water. Anesthesia was induced by intramuscular injection of midazolam (0.5 mg/kg), followed by ear vein injection of propofol (1.0 mg/kg). Anesthesia and analgesia were maintained by continuous infusion of sodium pentobarbital (8 mg/kg/h) and fentanyl (5 µg/kg/h). The average period of anesthesia was 72±2 h. A cuffed 6.5-mm endotracheal tube was advanced into the trachea. All animals were mechanically ventilated with a volume-controlled ventilator (Servo 900c; Siemens, Munich, Germany) with a tidal volume of 15 mL/kg, a ventilation rate of 12 to 20 breaths/min, a constant fraction of inspired oxygen of 0.21, and an inspiration/expiration ratio of 1∶2 with a positive end-expiratory pressure of 5 cmH_2_O. End-tidal PCO_2_ (ETCO_2_) was monitored with an in-line infrared capnograph (CO_2_SMOplus monitor, Respironics Inc., Pittsburgh, Pennsylvania, USA). Respiratory frequency was adjusted to maintain ETCO_2_ between 35 and 40 mmHg before inducing CA and after ROSC. Room temperature was maintained at 26°C. An angiographic catheter (7-Fr, Edwards Life Sciences, Irvine, California, USA) was inserted from the left femoral artery into the aortic arch for obtaining arterial blood samples and for measuring mean arterial pressure (MAP). Radiographic examination was used to verify placement of the angiographic catheter. The electrocardiogram and hemodynamic parameters such as heart rate (HR) and arterial pressure were electronically monitored (M1165; Hewlett-Packard Company, Palo Alto, California, USA) and recorded throughout the study. A 5-Fr catheter was inserted into the right external jugular vein to place an electrode catheter, and ventricular fibrillation (VF) was induced by a programmed electrical stimulation instrument (GY-600A; Kaifeng, Henan, China). All catheters were calibrated before use, and their positions were verified by the presence of typical pressure waves.

### Experimental Protocol

After preparation and catheter insertion, animals were allowed to equilibrate for 30 min to achieve a stable resting level, and baseline values were obtained. In 20 of the 24 pigs, VF was induced and was confirmed by the VF wave in the ECG and by sharply decreased blood pressure. When VF occurred, mechanical ventilation ceased. After 8 min of untreated VF, CPR was manually performed according to the 2010 American Heart Association Guidelines for Cardiopulmonary Resuscitation and Emergency Cardiovascular Care. After delivery of 30 compressions, animals received two breaths using a bag respirator with room air. After 2 min of CPR (compression-to-ventilation ratio of 30∶2), defibrillation (Smart Biphasic, Philips Healthcare, Eindhoven, Netherlands) was attempted using 150 J for the first attempt. If VF still persisted, another 2-min of CPR was resumed, followed by the first bolus of epinephrine (30 µg/kg). Additional doses of epinephrine were administered, if needed, every 3 min until ROSC was achieved. If the first defibrillation attempt was unsuccessful, 200 J were used for the second and all subsequent attempts. ROSC was defined as >10 min of systolic blood pressure maintained continuously >50 mmHg. Four animals with no ROSC after four attempts of defibrillation were pronounced dead. The remaining 16 successfully resuscitated pigs were randomly treated with either endovascular mild hypothermic (mild hypothermia group, MH, *n* = 8) or normothermic (control normothermia group, CN, *n* = 8) post-CA care. Another four miniature pigs were in the sham operation group (SO) with no VF, CPR, or mild hypothermia treatment. Randomization was performed using the envelope method. Controlled mild hypothermia was induced and maintained using an intravascular cooling device (CoolGard 3000, Alsius, Irvine, California, USA). Cooling (1.0°C/h) was initiated immediately after ROSC in the MH group. The heat-exchange catheter (Icy™, Alsius, Irvine, California, USA) was placed into the vena cava via the right external jugular vein, and cooled normal saline was infused through a closed loop system into two heat-exchange balloons located near the distal end of the catheter. The temperature of the normal saline was adjusted automatically by the CoolGard 3000 according to feedback to the external pump/refrigerant device from a microthermister attached to a Foley bladder catheter. Target temperature was set at 33°C and maintained for 12 h, followed by active rewarming to 38°C (0.5°C/h). Temperatures were measured via bladder-temperature probes and were recorded throughout the study. Animals underwent an intensive care period until 72 h after ROSC, and mechanical ventilation was resumed with the same settings as were used before CA. Ringer’s lactate and saline 0.9% or glucose were infused intravenously at a rate of 10 mL/kg/h to maintain fluid balance. At 72 h after ROSC, animals were sacrificed with an intravenous injection of propofol (60 mg) followed by an intravenous injection of potassium chloride (10 mL of 10 mol/L). Researchers were blinded to treatment until data collection was complete.

### Measurements

Blood samples were collected via arterial catheter at 6 time points: baseline and 0.5, 6, 12, 24, and 72 h after ROSC. The initial 5 mL of each blood sample was discarded. Blood samples (5 mL) were collected in 0.109 M trisodium citrate tubes (9∶1 vol/vol) and centrifuged immediately for 10 min at 3000 r/min. The plasma was used to measure prothrombin time (PT) and activated partial thromboplastin time (APTT) using reagents from Dade Behring Inc. (Westwood, Massachusetts, USA) and an automated blood coagulation analyzer (Sysmex CA-1500, Kobe, Japan). PT and APTT were measured at 37°C. Measurements were performed in triplicate, then averaged. The remaining plasma was stored at −80°C until analysis. Samples were analyzed by enzyme-linked immunosorbent assay (ELISA) using porcine ELISA kits (Groundwork Biotechnology Diagnosticate Ltd, San Diego, California, USA) for: antithrombin III (AT-III), protein C (PRO C), protein S (PRO S), thrombomodulin (TM), endothelial cell protein C receptor (EPCR), tissue factor pathway inhibitor (TFPI), tissue type plasminogen activator (tPA), plasminogen activator inhibitor 1 (PAI-1), and granule membrane protein α (GMP-140).

### Statistical Analysis

Statistical analysis was performed with SPSS 17.0 software (SPSS Inc., Chicago, Illinois, USA). All data were shown as mean ± standard deviation (SD), except for ROSC and survival numbers. Body weight, bladder temperature, mean arterial pressure, heart rate, duration of CPR and number of defibrillation attempts were compared between groups using a one-way analysis of variance (ANOVA). Coagulation, physiological anticoagulants, serum fibrinolysis and platelet activation parameters were compared between groups using a repeated- measures ANOVA. The Bonferroni test was used for multiple comparisons. A two sided *p*<0.05 was considered statistically significant.

## Results

### Baseline Characteristics, Resuscitation Data, and Bladder Temperature

At baseline, there were no significant group differences in body weight (BW), bladder temperature (BT), mean arterial pressure, or heart rate (*p*>0.05, Table 1). Duration of CPR (3.2±1.1 *vs*. 3.3±1.2 min, *p*>0.05) and number of defibrillation attempts (1.5±0.5 *vs*.1.7±0.6, *p*>0.05) were also similar between CN and MH groups.

**Table 1:** Baseline characteristics.

Group	BW (kg)	BT (°C)	MAP (mmHg)	HR (bpm)
CN (*n* = 8)	29.6±1.9	38.5±0.6	115±5	76±2
MH (*n* = 8)	29.6±2.2	38.5±0.5	113±5	76±4
SO (*n* = 4)	30.0±2.2	38.8±0.7	117±4	77±5

Values are mean ± SD. BW, body weight; BT, bladder temperature; MAP, mean arterial pressure; HR, heart rate; CN, control normothermia group; MH, mild hypothermia group; SO, sham operation group.

**Figure 1:**
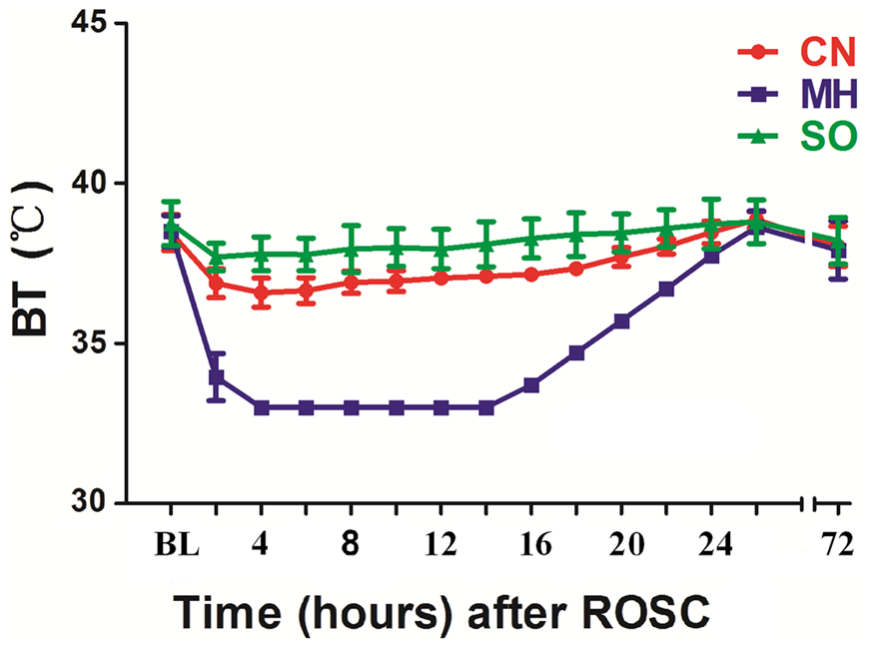
Bladder temperature. BT, bladder temperature; CN, control normothermia group; MH, mild hypothermia group; SO, sham operation group.

In the CN group, BT values were maintained at 36.5–38.8°C during the observation period. In the MH group, BT values decreased to the target temperature (33°C) within 2–4 h, remained at this temperature (33±0.2°C) for 12 h, and then gradually increased over 12 h (Figure 1), consistent with the landmark study by Bernard [6].

### Coagulation Parameters

**Figure 2:**
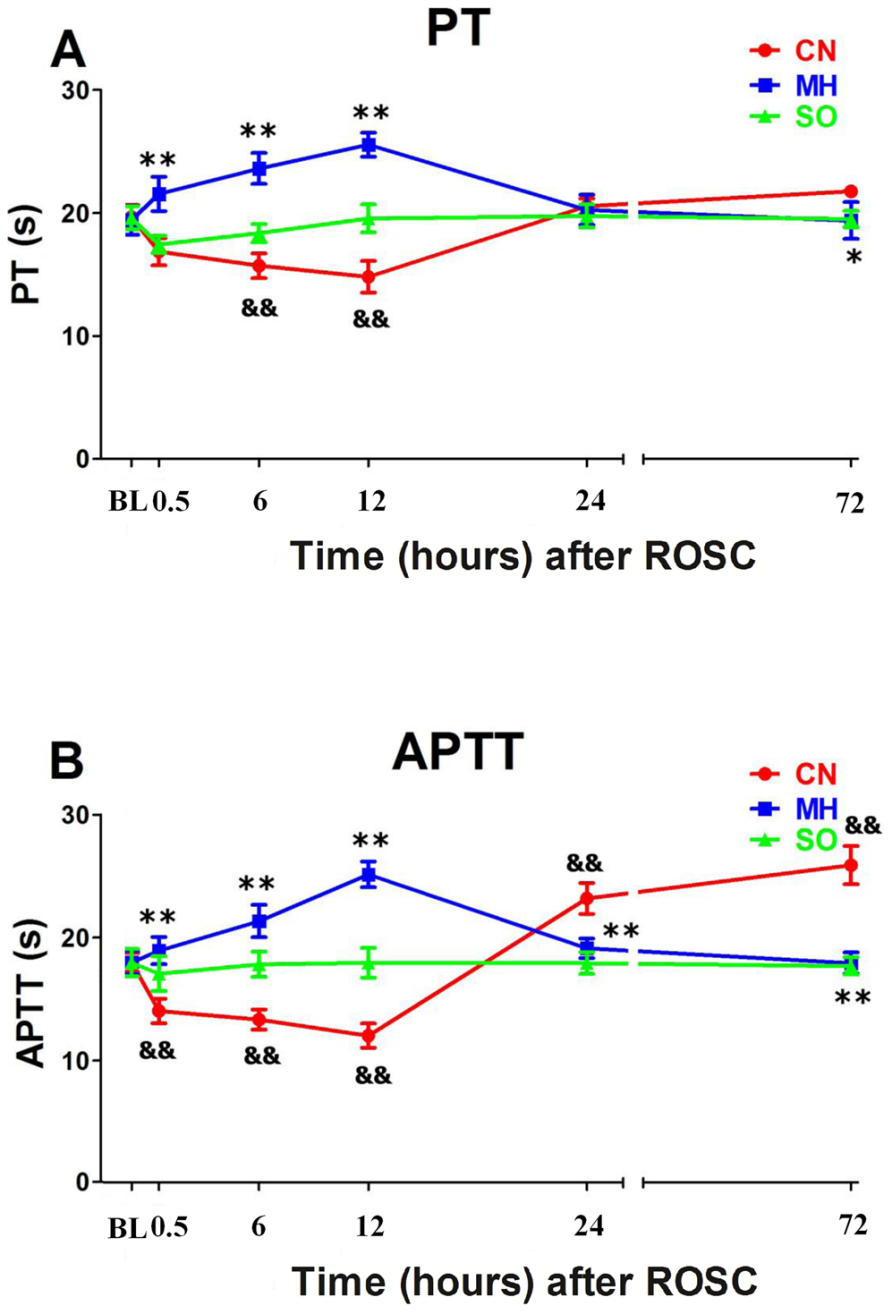
Coagulation parameters. (A) PT, prothrombin time; (B) APTT, activated partial thromboplastin time; CN, control normothermia group; MH, mild hypothermia group; SO, sham operation group.^ &&^ *p*<0.01 CN group *vs*. SO group.*p<0.05 MH group *vs*. CN group. **p<0.01 MH group *vs*. CN group.

The CN group had lower PT levels from 6 to 12 h and lower APTT levels from 0.5 to 12 h after ROSC, compared to the SO group (all *p*<0.01). PT returned to SO levels at 24 and 72 h, while APTT levels were elevated at 24 and 72 h, compared to the SO group (all *p*<0.01). Compared to the CN group, the MH group showed increased PT (Figure 2A) and APTT (Figure 2B) from 0.5 to 12 h after ROSC (all *p*<0.01). PT levels were similar at 24 h, but decreased at 72 h compared to the CN group, and APTT levels were decreased at 24 and 72 h compared to the CN group (all *p*<0.01).

### Physiological Anticoagulants

**Figure 3:**
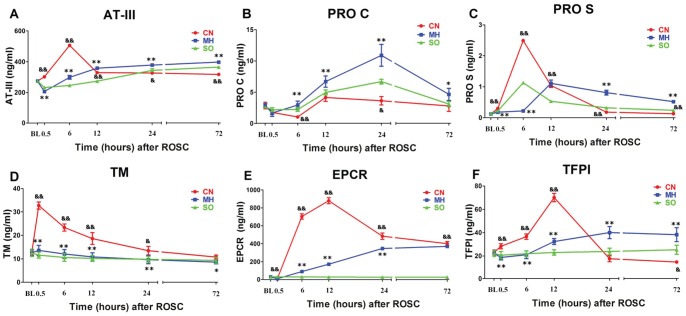
Serum physiological anticoagulant levels. (A) AT-III, Antithrombin III; (B) PRO C, protein C; (C) PRO S, protein S; (D) TM, thrombomodulin; (E) EPCR, endothelial cell protein C receptor; (F) TFPI, tissue factor pathway inhibitor; CN, control normothermia group; MH, mild hypothermia group; SO, sham operation group.^ &^ *p*<0.05 CN group vs. SO group. ^&&^ *p*<0.01 CN group vs. SO group. *p<0.05 MH group vs. CN group. **p<0.01 MH group vs. CN group.

As shown in Figure 3, compared to the SO group, the CN group had lower serum levels of PRO C at 6 h (*p*<0.01) and higher serum levels of AT-III, PRO S, TM, EPCR and TFPI at 6 and 12 h after ROSC (all *p*<0.01). At 24 h, the CN group had decreased AT-III, PRO S, and PRO C (all *p*<0.05) and increased TM and EPCR (all *p*<0.05). At 72 h, the CN group had decreased AT-III, PRO S and TFPI (all *p*<0.05) and increased EPCR (*p*<0.01).

Compared to the CN group, at 6 h after ROSC, the MH group had decreased serum levels of AT-III, PRO S, TM, EPCR and TFPI (all *p*<0.01). At 12 h, the MH group had decreased TM, EPCR and TFPI and increased AT-III (all *p*<0.01). At 24 h, the MH group had increased AT-III, PRO S and TFPI (all *p*<0.01) and decreased TM and EPCR (all *p*<0.01). At 72 h, the MH group had increased AT-III, PRO S and TFPI (all *p*<0.01) and decreased TM (*p*<0.01). PRO C was consistently increased from 6 to 72 h after ROSC (all *p*<0.05).

### Serum Fibrinolysis and Platelet Activation Parameters

**Figure 4:**
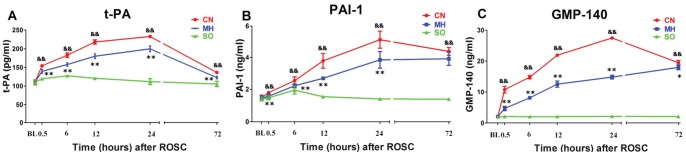
Serum fibrinolysis and platelet activation parameter levels. (A) tPA, tissue type plasminogen activator; (B) PAI-1, plasminogen activator inhibitor 1; (C) GMP-140, granule membrane protein α; CN, control normothermia group; MH, mild hypothermia group; SO, sham operation group.^ &&^ *p*<0.01 CN group vs. SO group. *p<0.05 MH group vs. CN group. **p<0.01 MH group vs. CN group.

As shown in Figure 4, compared to the SO group, the CN group had higher serum levels of tPA, PAI-1, and GMP-140 from 6 to 72 h after ROSC (all *p*<0.01). Compared to the CN group, the MH group had decreases in these parameters from 6 to 72 h after ROSC (except for PAI-1 at 72 h) (all *p*<0.05).

## Discussion

The key findings of this study were: (1) whole-body mild hypothermia attenuated the impairment in blood coagulation at 72 after ROSC, despite contributing to impairment of blood coagulation during cooling; (2) whole-body mild hypothermia increased serum levels of physiological anticoagulants, such as PRO C and AT-III, and decreased EPCR and TFPI during cooling; (3) whole-body mild hypothermia inhibited fibrinolysis and platelet activation during cooling and after rewarming; and (4) slow rewarming had no obvious adverse effects on coagulation-fibrinolysis, physiological anticoagulants, or platelet activation parameters.

CA and resuscitation are accompanied by marked activation of blood coagulation, which can induce microthrombus and blockage of microcirculation in the brain or myocardium, leading to ischemia-reperfusion injury [4], [5], [12], [13], [14]. In the well-established pig CA model induced by VF [11], [15], [16], [17], [18], [19], [20], [21], [22], [23], we found that whole-body mild hypothermia prolonged APTT and PT, consistent with a previous report [24]. In addition, some studies have demonstrated that mild hypothermia resulted in mild platelet dysfunction, partial inhibition of the coagulation cascade, and decreased platelet count [9], [25], [26], [27]. Thus, the findings from our laboratory and from others suggest that mild hypothermia exerts an anticoagulant effect. Suppressing coagulation may provide protection by decreasing cerebral or myocardial microthrombus formation [24], [27]. In addition, increasing evidence has shown a crosstalk between inflammation and thrombosis [28], [29], [30] and a concurrent activation of the inflammatory system and the coagulation cascade during CPR and after ROSC [31] [32]. Thus, the effects of mild hypothermia on coagulation are also associated with inhibition of inflammation [15], [33], [34].

Our data showed that mild hypothermia significantly increased serum levels of physiological anticoagulants, such as PRO C during cooling and AT-III at 12 h after ROSC, which could explain its anticoagulant effects. There are three major anticoagulant mechanisms in the coagulation cascade: 1) Antithrombin system: AT-III is a key inhibitor of the coagulation system, which directly inhibits activated thrombin through formation of the thrombin-antithrombin complex. 2) Protein C system: PRO C is activated on the cell surface when thrombin binds to thrombomodulin (TM). The conversion to activated protein C (APC) is augmented by EPCR, which is present on endothelial cells. PRO S, as the cofactor of PRO C, could significantly enhance PRO C function. 3) TFPI: TFPI is a Kunitz-type inhibitor that directly inhibits tissue factor/factor VIIa complex in the presence of factor Xa, as the specific inhibitor of the extrinsic coagulation pathway. However, our study also demonstrated that mild hypothermia decreased serum levels of AT-III and PRO S at 0.5 and 6 h after ROSC and TM, EPCR and TFPI during cooling. TM and EPCR are markers of endothelial injury [14], [35], thus decreased levels of these substances during cooling suggests that mild hypothermia may protect endothelial cells, consistent with the study by Kazanskaya [36]. We speculate that protection against endothelial cell damage by mild hypothermia might contribute to the increased levels of PRO C observed during cooling. In addition, the pattern of effects of mild hypothermia on physiological anticoagulants might indicate a new equilibrium that prevents dysfunctional anticoagulation during cooling.

The coagulation cascade is now recognized to be a series of proteolytic events that are primarily localized to the surface of activated platelets. Once platelets become activated by exposure to activated endothelium, they release mediators such as GMP-140 (namely P-selectin) [37], [38], which promotes microvesicle formation and platelet adherence, enabling the cascading proteolytic cleavages of zymogens to active enzymes and culminating in thrombin generation. In this study, we observed increased GMP-140 in normothermic pigs resuscitated from CA, consistent with the clinical study by Bottiger [39], whereas mild hypothermia attenuated this increase in GMP-140. These data suggest that mild hypothermia may exert its anticoagulant effects by inhibiting platelet activation.

In addition to coagulation/anticoagulation, fibrinolysis/antifibrinolysis systems are activated in patients who undergo CPR [4], [10], [40]. In the present study, normothermic pigs resuscitated from CA had increased levels of tissue plasminogen activator (tPA), which catalyzes the conversion of plasminogen to plasmin (responsible for clot breakdown) and of plasminogen activator inhibitor-1 (PAI-1), which is the principal inhibitor of tPA. Bottiger et al. [4] observed only slight to moderate activation of endogenous fibrinolysis, which could not adequately balance the marked activation of blood coagulation. They concluded that these changes may contribute to reperfusion disorders, such as the cerebral “no-reflow” phenomenon, by inducing fibrin deposition and formation of microthrombi [4]. However, our results showed that mild hypothermia significantly decreased tPA and PAI-1 levels, indicating inhibition of activated fibrinolysis/antifibrinolysis in addition to blood coagulation, consistent with the study by Staikou [41].

Interestingly, we found that mild hypothermia attenuated blood coagulation impairment despite increases in AT-III, PRO C, PRO S and TFPI at 72 h after ROSC, which might partially explain its beneficial effects. We speculate that the reduction in blood coagulation impairment after treatment by mild hypothermia might be associated with other positive effects of mild hypothermia, whose mechanisms need to be further investigated. Another interesting finding was that slow rewarming had no obvious adverse effects on coagulation-fibrinolysis, physiological anticoagulants, or platelet activation. At the end of rewarming (24 h after ROSC), compared to the CN group, the MH group showed similar PT, decreased APTT, tPA, PAI-1 and GMP-140, and increased AT-III, PRO S and TFPI, despite decreased TM and EPCR.

The current study had some limitations. First, catheter insertion may itself affect coagulation conditions within animals. However, we designed the SO group and strictly standardized the experimental protocol to minimize confounding factors. Second, the experiment was performed on apparently healthy pigs, whereas most individuals with CA have underlying pathologic findings. Third, this study did not include blood gas analysis to assess acid base status. Finally, high tidal volume (15 ml/kg), which was used in this study to maintain normocapnia, may induce lung injury with subsequent inflammation. However, all animals were ventilated using the same procedure, regardless of their group assignment.

In conclusion, mild hypothermia exerted an anticoagulant effect during cooling, which may have inhibitory effects on microthrombus formation. Furthermore, mild hypothermia inhibited fibrinolysis and platelet activation during cooling and attenuated blood coagulation impairment after rewarming. Slow rewarming had no obvious adverse effects on blood coagulation.
